# *N*-(4-Chloro­phen­yl)-4-nitro­benzene­sulfonamide

**DOI:** 10.1107/S1600536812049070

**Published:** 2012-12-05

**Authors:** U. Chaithanya, Sabine Foro, B. Thimme Gowda

**Affiliations:** a Department of Chemistry, Mangalore University, Mangalagangotri 574 199, Mangalore, India; b Institute of Materials Science, Darmstadt University of Technology, Petersenstrasse 23, D-64287 Darmstadt, Germany

## Abstract

In the title compound, C_12_H_9_ClN_2_O_4_S, the dihedral angle between the benzene rings is 31.4 (2)°. In the crystal, N—H⋯O hydrogen bonds link the mol­ecules into *C*(4) chains running along the *a-*axis direction.

## Related literature

For our studies on the effects of substituents on the structures and other aspects of *N*-(ar­yl)-amides, see: Gowda & Weiss (1994 ▶), of *N*-aryl­sulfonamides, see: Chaithanya *et al.* (2012 ▶); Gowda *et al.* (2003 ▶) and of *N*-chloro­aryl­sulfonamides, see: Gowda *et al.* (2005 ▶); Shetty & Gowda (2004 ▶).
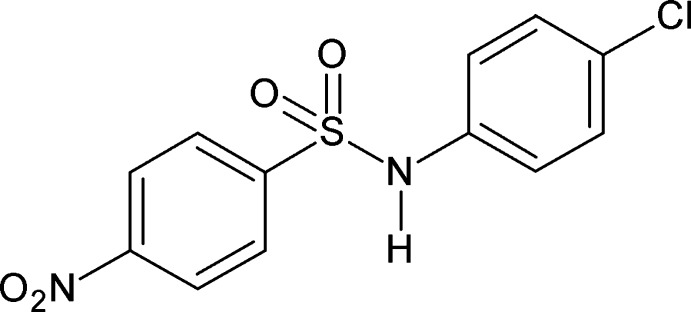


## Experimental

### 

#### Crystal data

C_12_H_9_ClN_2_O_4_S
*M* *_r_* = 312.72
Monoclinic, 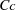

*a* = 5.0881 (4) Å
*b* = 13.0313 (9) Å
*c* = 19.886 (2) Å
β = 94.194 (7)°
*V* = 1315.00 (19) Å^3^
*Z* = 4
Mo *K*α radiation
μ = 0.46 mm^−1^
*T* = 293 K
0.46 × 0.24 × 0.12 mm

#### Data collection

Oxford Diffraction Xcalibur diffractometer with a Sapphire CCD detector
Absorption correction: multi-scan (*CrysAlis RED*; Oxford Diffraction, 2009 ▶) *T*_min_ = 0.815, *T*_max_ = 0.947
2432 measured reflections
1557 independent reflections
1432 reflections with *I* > 2σ(*I*)
*R*_int_ = 0.014

#### Refinement

*R*[*F* ^2^ > 2σ(*F* ^2^)] = 0.041
*wR*(*F* ^2^) = 0.092
*S* = 1.13
1557 reflections
184 parameters
3 restraints
H atoms treated by a mixture of independent and constrained refinement
Δρ_max_ = 0.29 e Å^−3^
Δρ_min_ = −0.29 e Å^−3^
Absolute structure: Flack (1983 ▶), 203 Friedel pairs
Flack parameter: −0.02 (11)

### 

Data collection: *CrysAlis CCD* (Oxford Diffraction, 2009 ▶); cell refinement: *CrysAlis CCD*; data reduction: *CrysAlis RED* (Oxford Diffraction, 2009 ▶); program(s) used to solve structure: *SHELXS97* (Sheldrick, 2008 ▶); program(s) used to refine structure: *SHELXL97* (Sheldrick, 2008 ▶); molecular graphics: *PLATON* (Spek, 2009 ▶); software used to prepare material for publication: *SHELXL97*.

## Supplementary Material

Crystal structure: contains datablock(s) I, global. DOI: 10.1107/S1600536812049070/bt6873sup1.cif

Structure factors: contains datablock(s) I. DOI: 10.1107/S1600536812049070/bt6873Isup2.hkl

Supplementary material file. DOI: 10.1107/S1600536812049070/bt6873Isup3.cml

Additional supplementary materials:  crystallographic information; 3D view; checkCIF report

## Figures and Tables

**Table 1:** Hydrogen-bond geometry (Å, °)

*D*—H⋯*A*	*D*—H	H⋯*A*	*D*⋯*A*	*D*—H⋯*A*
N1—H1*N*⋯O2^i^	0.85 (2)	2.16 (2)	3.007 (4)	173 (5)

Symmetry code: (i) 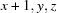.

**Fig. 1.**
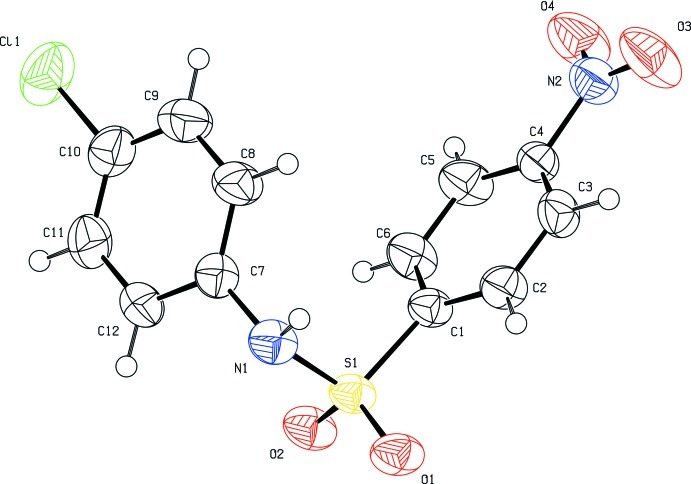
Molecular structure of the title compound, showing the atom labelling scheme and with displacement ellipsoids drawn at the 50% probability level.

**Fig. 2.**
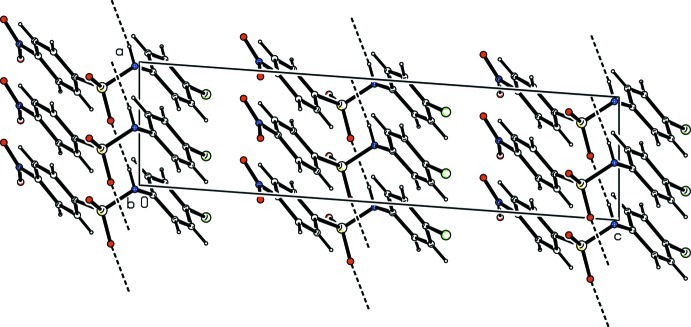
Molecular packing of the title compound with hydrogen bonding shown as dashed lines.

## supplementary crystallographic information

### Comment

As a part of our studies on the substituent effects on the structures and other aspects of *N*-(aryl)-amides (Gowda & Weiss, 1994); *N*-arylsulfonamides (Chaithanya *et al.*, 2012; Gowda *et al.*, 2003) and *N*-chloroarylsulfonamides (Gowda *et al.*, 2005; Shetty & Gowda, 2004), in the present work, the crystal structure of *N*-(4-chlorophenyl)-4-nitrobenzenesulfonamide (I) has been determined (Fig. 1).

The conformation of the N—C bond in the —SO_2_—NH—C segment has *gauche* torsions with respect to the S═O bonds (Fig. 1), similar to that observed in *N*-(phenyl)-4-nitrobenzenesulfonamide (II) (Chaithanya *et al.*, 2012). The molecule is twisted at the S—N bond with the torsional angle of 63.74 (35)°, compared to the value of 61.89 (32)° in (II).

The dihedral angle between the sulfonyl and the anilino rings is 31.40 (23)°, compared to the value of 36.19 (18)° in (II).

In the crystal, intermolecular N—H···O hydrogen bond interactions link the molecules into C(4) chains. Part of the crystal structure is shown in Fig. 2.

### Experimental

The title compound was prepared by treating 4-nitrobenzenesulfonylchloride with 4-chloroaniline in the stoichiometric ratio and boiling the reaction mixture for 15 minutes. The reaction mixture was then cooled to room temperature and added to ice cold water (100 ml). The resultant solid *N*-(4-chlorophenyl)-4-nitrobenzenesulfonamide was filtered under suction and washed thoroughly with cold water and dilute HCl to remove the excess sulfonylchloride and aniline, respectively. It was then recrystallized from dilute ethanol. The purity of the compound was checked and characterized by its infrared spectra.

Prism like colourless single crystals of the title compound used in X-ray diffraction studies were grown in an ethanolic solution by slow evaporation of the solvent at room temperature.

### Refinement

H atoms bonded to C were positioned with idealized geometry using a riding model with the aromatic C—H = 0.93 Å. The amino H atom was freely refined with the N—H distance restrained to 0.86 (2) Å. All H atoms were refined with isotropic displacement parameters set at 1.2 *U*_eq_ of the parent atom.

### Crystal data

**Table:**

C_12_H_9_ClN_2_O_4_S	*F*(000) = 640
*M**_r_* = 312.72	*D*_x_ = 1.580 Mg m^−^^3^
Monoclinic, *C**c*	Mo *K*α radiation, λ = 0.71073 Å
Hall symbol: C -2yc	Cell parameters from 1307 reflections
*a* = 5.0881 (4) Å	θ = 2.6–27.8°
*b* = 13.0313 (9) Å	µ = 0.46 mm^−^^1^
*c* = 19.886 (2) Å	*T* = 293 K
β = 94.194 (7)°	Prism, colourless
*V* = 1315.00 (19) Å^3^	0.46 × 0.24 × 0.12 mm
*Z* = 4	

### Data collection

**Table:**

Oxford Diffraction Xcalibur diffractometer with a Sapphire CCD detector	1557 independent reflections
Radiation source: fine-focus sealed tube	1432 reflections with *I* > 2σ(*I*)
Graphite monochromator	*R*_int_ = 0.014
Rotation method data acquisition using ω scans	θ_max_ = 26.4°, θ_min_ = 3.1°
Absorption correction: multi-scan (*CrysAlis RED*; Oxford Diffraction, 2009)	*h* = −6→3
*T*_min_ = 0.815, *T*_max_ = 0.947	*k* = −8→16
2432 measured reflections	*l* = −24→24

### Refinement

**Table:**

Refinement on *F*^2^	Secondary atom site location: difference Fourier map
Least-squares matrix: full	Hydrogen site location: inferred from neighbouring sites
*R*[*F*^2^ > 2σ(*F*^2^)] = 0.041	H atoms treated by a mixture of independent and constrained refinement
*wR*(*F*^2^) = 0.092	*w* = 1/[σ^2^(*F*_o_^2^) + (0.0318*P*)^2^ + 1.8686*P*] where *P* = (*F*_o_^2^ + 2*F*_c_^2^)/3
*S* = 1.13	(Δ/σ)_max_ < 0.001
1557 reflections	Δρ_max_ = 0.29 e Å^−^^3^
184 parameters	Δρ_min_ = −0.29 e Å^−^^3^
3 restraints	Absolute structure: Flack (1983), 203 Friedel pairs
Primary atom site location: structure-invariant direct methods	Flack parameter: −0.02 (11)

### Special details

**Table:**

Experimental. Absorption correction: CrysAlis RED (Oxford Diffraction, 2009) Empirical absorption correction using spherical harmonics, implemented in SCALE3 ABSPACK scaling algorithm.
Geometry. All e.s.d.'s (except the e.s.d. in the dihedral angle between two l.s. planes) are estimated using the full covariance matrix. The cell e.s.d.'s are taken into account individually in the estimation of e.s.d.'s in distances, angles and torsion angles; correlations between e.s.d.'s in cell parameters are only used when they are defined by crystal symmetry. An approximate (isotropic) treatment of cell e.s.d.'s is used for estimating e.s.d.'s involving l.s. planes.
Refinement. Refinement of *F*^2^ against ALL reflections. The weighted *R*-factor *wR* and goodness of fit *S* are based on *F*^2^, conventional *R*-factors *R* are based on *F*, with *F* set to zero for negative *F*^2^. The threshold expression of *F*^2^ > σ(*F*^2^) is used only for calculating *R*-factors(gt) *etc*. and is not relevant to the choice of reflections for refinement. *R*-factors based on *F*^2^ are statistically about twice as large as those based on *F*, and *R*- factors based on ALL data will be even larger.

### Fractional atomic coordinates and isotropic or equivalent isotropic displacement parameters (Å^2^)

**Table:**

	*x*	*y*	*z*	*U*_iso_*/*U*_eq_	
C1	0.3399 (8)	0.8673 (3)	0.3654 (2)	0.0386 (9)	
C2	0.5320 (9)	0.8525 (3)	0.3209 (2)	0.0464 (11)	
H2	0.6101	0.7885	0.3173	0.056*	
C3	0.6079 (10)	0.9336 (3)	0.2819 (2)	0.0479 (12)	
H3	0.7365	0.9249	0.2514	0.057*	
C4	0.4895 (9)	1.0268 (3)	0.2889 (2)	0.0405 (10)	
C5	0.2944 (10)	1.0418 (3)	0.3320 (3)	0.0522 (12)	
H5	0.2142	1.1056	0.3350	0.063*	
C6	0.2198 (9)	0.9608 (3)	0.3704 (3)	0.0499 (11)	
H6	0.0878	0.9694	0.3999	0.060*	
C7	0.4286 (8)	0.8807 (4)	0.5281 (2)	0.0417 (10)	
C8	0.5769 (10)	0.9675 (4)	0.5181 (3)	0.0549 (12)	
H8	0.7061	0.9655	0.4873	0.066*	
C9	0.5366 (12)	1.0564 (4)	0.5527 (3)	0.0635 (15)	
H9	0.6395	1.1141	0.5464	0.076*	
C10	0.3403 (11)	1.0586 (4)	0.5970 (2)	0.0539 (13)	
C11	0.1964 (11)	0.9734 (4)	0.6082 (2)	0.0584 (14)	
H11	0.0676	0.9757	0.6390	0.070*	
C12	0.2400 (10)	0.8829 (4)	0.5740 (2)	0.0535 (12)	
H12	0.1425	0.8243	0.5821	0.064*	
N1	0.4632 (7)	0.7883 (3)	0.4904 (2)	0.0454 (9)	
H1N	0.620 (5)	0.783 (4)	0.479 (2)	0.055*	
N2	0.5752 (9)	1.1153 (3)	0.2495 (2)	0.0540 (10)	
O1	0.3503 (6)	0.6725 (2)	0.39544 (17)	0.0528 (8)	
O2	0.0062 (6)	0.7821 (2)	0.44006 (16)	0.0508 (8)	
O3	0.7655 (9)	1.1042 (3)	0.2172 (2)	0.0846 (14)	
O4	0.4539 (9)	1.1952 (3)	0.2524 (2)	0.0773 (13)	
Cl1	0.2766 (3)	1.17181 (11)	0.63901 (8)	0.0816 (5)	
S1	0.27032 (19)	0.76820 (7)	0.42212 (6)	0.0408 (3)	

### Atomic displacement parameters (Å^2^)

**Table:**

	*U*^11^	*U*^22^	*U*^33^	*U*^12^	*U*^13^	*U*^23^
C1	0.035 (2)	0.034 (2)	0.047 (2)	−0.0002 (18)	0.0082 (19)	0.0000 (18)
C2	0.049 (3)	0.035 (2)	0.057 (3)	0.004 (2)	0.017 (2)	−0.005 (2)
C3	0.052 (3)	0.048 (3)	0.046 (2)	0.003 (2)	0.019 (2)	−0.002 (2)
C4	0.043 (2)	0.038 (2)	0.042 (2)	−0.002 (2)	0.009 (2)	0.0015 (18)
C5	0.054 (3)	0.032 (2)	0.072 (3)	0.010 (2)	0.016 (3)	0.005 (2)
C6	0.043 (3)	0.043 (2)	0.066 (3)	0.007 (2)	0.023 (2)	0.001 (2)
C7	0.035 (2)	0.046 (3)	0.044 (2)	0.003 (2)	0.0044 (19)	−0.001 (2)
C8	0.049 (3)	0.053 (3)	0.066 (3)	−0.010 (2)	0.021 (2)	−0.005 (2)
C9	0.075 (4)	0.045 (3)	0.073 (4)	−0.011 (3)	0.017 (3)	−0.001 (2)
C10	0.070 (3)	0.042 (3)	0.049 (3)	0.011 (2)	0.000 (3)	−0.002 (2)
C11	0.057 (3)	0.069 (4)	0.051 (3)	0.000 (3)	0.021 (2)	−0.008 (2)
C12	0.060 (3)	0.053 (3)	0.050 (3)	−0.011 (2)	0.020 (2)	0.000 (2)
N1	0.0347 (19)	0.045 (2)	0.058 (2)	0.0038 (17)	0.0116 (17)	−0.0014 (17)
N2	0.061 (3)	0.045 (2)	0.058 (2)	0.000 (2)	0.015 (2)	0.0051 (18)
O1	0.055 (2)	0.0328 (15)	0.072 (2)	0.0009 (14)	0.0165 (17)	−0.0057 (14)
O2	0.0334 (17)	0.0510 (19)	0.070 (2)	−0.0034 (14)	0.0145 (16)	0.0022 (15)
O3	0.087 (3)	0.069 (3)	0.106 (3)	0.008 (2)	0.057 (3)	0.022 (2)
O4	0.091 (3)	0.0454 (19)	0.100 (3)	0.013 (2)	0.038 (3)	0.0190 (19)
Cl1	0.1210 (14)	0.0577 (8)	0.0657 (9)	0.0210 (9)	0.0044 (9)	−0.0131 (7)
S1	0.0349 (5)	0.0353 (5)	0.0534 (6)	−0.0006 (5)	0.0110 (4)	−0.0006 (5)

### Geometric parameters (Å, º)

**Table:**

C1—C6	1.370 (6)	C8—C9	1.370 (7)
C1—C2	1.378 (6)	C8—H8	0.9300
C1—S1	1.768 (4)	C9—C10	1.381 (7)
C2—C3	1.383 (6)	C9—H9	0.9300
C2—H2	0.9300	C10—C11	1.357 (7)
C3—C4	1.368 (6)	C10—Cl1	1.737 (5)
C3—H3	0.9300	C11—C12	1.387 (7)
C4—C5	1.371 (6)	C11—H11	0.9300
C4—N2	1.479 (6)	C12—H12	0.9300
C5—C6	1.374 (6)	N1—S1	1.637 (4)
C5—H5	0.9300	N1—H1N	0.85 (2)
C6—H6	0.9300	N2—O3	1.209 (5)
C7—C12	1.373 (6)	N2—O4	1.214 (5)
C7—C8	1.382 (7)	O1—S1	1.426 (3)
C7—N1	1.436 (6)	O2—S1	1.427 (3)
			
C6—C1—C2	120.8 (4)	C8—C9—C10	118.7 (5)
C6—C1—S1	119.4 (3)	C8—C9—H9	120.7
C2—C1—S1	119.5 (3)	C10—C9—H9	120.7
C1—C2—C3	119.6 (4)	C11—C10—C9	120.8 (5)
C1—C2—H2	120.2	C11—C10—Cl1	119.6 (4)
C3—C2—H2	120.2	C9—C10—Cl1	119.6 (4)
C4—C3—C2	118.5 (4)	C10—C11—C12	120.5 (4)
C4—C3—H3	120.8	C10—C11—H11	119.7
C2—C3—H3	120.8	C12—C11—H11	119.7
C3—C4—C5	122.4 (4)	C7—C12—C11	119.2 (4)
C3—C4—N2	119.3 (4)	C7—C12—H12	120.4
C5—C4—N2	118.3 (4)	C11—C12—H12	120.4
C4—C5—C6	118.7 (4)	C7—N1—S1	118.6 (3)
C4—C5—H5	120.7	C7—N1—H1N	111 (4)
C6—C5—H5	120.7	S1—N1—H1N	106 (4)
C1—C6—C5	120.0 (4)	O3—N2—O4	123.9 (4)
C1—C6—H6	120.0	O3—N2—C4	117.8 (4)
C5—C6—H6	120.0	O4—N2—C4	118.3 (4)
C12—C7—C8	119.6 (4)	O1—S1—O2	120.2 (2)
C12—C7—N1	118.9 (4)	O1—S1—N1	106.2 (2)
C8—C7—N1	121.5 (4)	O2—S1—N1	106.9 (2)
C9—C8—C7	121.1 (4)	O1—S1—C1	109.0 (2)
C9—C8—H8	119.5	O2—S1—C1	107.7 (2)
C7—C8—H8	119.5	N1—S1—C1	106.1 (2)
			
C6—C1—C2—C3	1.2 (7)	N1—C7—C12—C11	−176.8 (5)
S1—C1—C2—C3	−172.7 (4)	C10—C11—C12—C7	−0.7 (8)
C1—C2—C3—C4	0.4 (7)	C12—C7—N1—S1	83.9 (5)
C2—C3—C4—C5	−1.7 (8)	C8—C7—N1—S1	−94.8 (5)
C2—C3—C4—N2	177.7 (4)	C3—C4—N2—O3	−7.2 (7)
C3—C4—C5—C6	1.5 (8)	C5—C4—N2—O3	172.3 (5)
N2—C4—C5—C6	−177.9 (5)	C3—C4—N2—O4	174.2 (5)
C2—C1—C6—C5	−1.4 (7)	C5—C4—N2—O4	−6.3 (7)
S1—C1—C6—C5	172.5 (4)	C7—N1—S1—O1	179.6 (3)
C4—C5—C6—C1	0.0 (8)	C7—N1—S1—O2	−50.9 (4)
C12—C7—C8—C9	−0.8 (8)	C7—N1—S1—C1	63.7 (3)
N1—C7—C8—C9	177.8 (5)	C6—C1—S1—O1	162.6 (4)
C7—C8—C9—C10	−1.3 (9)	C2—C1—S1—O1	−23.4 (4)
C8—C9—C10—C11	2.5 (9)	C6—C1—S1—O2	30.7 (4)
C8—C9—C10—Cl1	−177.5 (4)	C2—C1—S1—O2	−155.3 (4)
C9—C10—C11—C12	−1.5 (8)	C6—C1—S1—N1	−83.4 (4)
Cl1—C10—C11—C12	178.5 (4)	C2—C1—S1—N1	90.5 (4)
C8—C7—C12—C11	1.9 (8)		

### Hydrogen-bond geometry (Å, º)

**Table:**

*D*—H···*A*	*D*—H	H···*A*	*D*···*A*	*D*—H···*A*
N1—H1*N*···O2^i^	0.85 (2)	2.16 (2)	3.007 (4)	173 (5)

Symmetry code: (i) *x*+1, *y*, *z*.
